# Humanistic and Economic Burden of Patients With Keratoconus

**DOI:** 10.7759/cureus.106633

**Published:** 2026-04-08

**Authors:** Maria Dionysopoulou, Jason Makripoulias, Nikos Kotsopoulos, Eleftheria Karampli, Dimitrios S Papaconstantinou, Marilita M Moschos, Vasiliki Efthymiou, Konstantinos Droutsas

**Affiliations:** 1 First Department of Ophthalmology, School of Medicine, National and Kapodistrian University of Athens, Athens, GRC; 2 Department of Economics/Health Economics, National and Kapodistrian University of Athens, Athens, GRC; 3 Laboratory for Health Technology Assessment, Department of Public Health Policy, School of Public Health, University of West Attica, Athens, GRC; 4 Center for Adolescent Medicine and UNESCO Chair in Adolescence Health Care, First Department of Pediatrics, School of Medicine, National and Kapodistrian University of Athens, Athens, GRC

**Keywords:** economic burden, keratoconus (kc), presenteeism, vision-related quality of life, work productivity loss

## Abstract

Background and objective

Keratoconus is a chronic, progressive corneal disease that occurs primarily in people of working age and significantly affects visual function. However, its broader impact on vision-related quality of life (VRQoL), work productivity, and functional capacity remains poorly investigated. The primary objective of this study was to assess the impact of keratoconus on VRQoL and work productivity. Secondary objectives included the evaluation of daily activity impairment and the investigation of associations between these outcomes and demographic, clinical, and economic factors.

Methods

A cross-sectional study was conducted in patients with keratoconus, who completed the National Eye Institute Visual Function Questionnaire-25 (VFQ-25) and the Work Productivity and Activity Impairment (WPAI) Questionnaire. Demographic and clinical data, including Amsler-Krumeich staging and type of treatment, were collected. Associations were assessed using univariate and multivariate analyses.

Results

Reduced VRQoL was significantly associated with increased presenteeism, overall work productivity loss, and functional disability. The VFQ-25 subscales for near and distance vision activities, mental health, and caregiver dependence showed the strongest associations. Higher age, advanced stages of keratoconus, especially in the worse eye, and surgical treatment were associated with lower VRQoL. No independent association was observed between VRQoL and annual treatment costs.

Conclusions

Keratoconus imposes a substantial functional and psychosocial burden, significantly impairing VRQoL and work productivity. Reduced VRQoL was strongly associated with increased presenteeism, overall productivity loss, and activity impairment, highlighting its role as a key indicator of disease burden. Disease severity, older age, and surgical treatment were identified as important determinants of poorer outcomes. These findings underscore the importance of early detection and comprehensive, patient-centered management strategies, including the routine assessment of VRQoL and functional outcomes in clinical practice.

## Introduction

Keratoconus is a bilateral, noninfectious, and noninflammatory corneal disorder characterized by progressive thinning and protrusion of the corneal stroma, leading to a conical corneal shape. It typically begins in late childhood or early adulthood and may progress until the fourth decade of life. The condition primarily affects the central and inferior paracentral regions of the cornea, causing irregular astigmatism and myopic shift, which result in distorted and reduced vision. As the disease progresses, visual correction with spectacles or contact lenses may become inadequate, and advanced cases may develop complications such as corneal hydrops or scarring requiring surgical intervention. Although the exact cause remains unknown, advances in corneal topography and tomography have improved early detection, contributing to the increased reported prevalence of keratoconus worldwide [1].

The prevalence of keratoconus worldwide ranges from 0.02% to 0.67%, with this variation attributed to differences in study design, heterogeneity, and definitions and classification of keratoconus [2]. In addition, most study samples have been collected from patients visiting public health facilities, mainly hospital ophthalmology clinics, or from insurance company data, which may underestimate the true incidence of the disease, as patients are often asymptomatic and early-onset or subtle forms may not be detected [3-5]. The incidence of keratoconus has been found to vary depending on geographical location, with countries in Northern Europe, the Urals in Russia, North America, and Japan showing a low incidence, while in Middle Eastern countries, including India and China, the frequency of cases increases [6,7]. A recent meta-analysis including more than 50 million people from 15 countries found that the global incidence of keratoconus was 138 per 100,000 [8].

The risk factors underlying keratoconus remain incompletely understood; however, a strong genetic and hereditary component has been consistently demonstrated [6]. A significant association with positive family history and consanguinity has been reported, with 10% of affected individuals presenting familial predisposition compared to 0.05% of controls [9,10]. Environmental influences have also been implicated, particularly low relative humidity, which has been associated with increased disease prevalence, whereas other climatic parameters showed no significant correlation after adjustment [2]. Ethnic and socioeconomic disparities appear to influence disease severity at presentation, with certain populations demonstrating more advanced clinical findings at diagnosis [11,12]. Eye rubbing and atopy represent important and potentially modifiable risk factors [13,14]. Additionally, endogenous hormonal fluctuations and exogenous hormone use have been associated with structural and functional corneal alterations, suggesting a possible role of hormonal regulation in disease progression [15-19].

Regarding the average age of diagnosis, a study in Western India reported 20.5 years [20], while in Israel and Saudi Arabia, the age of onset was lower: 18.4 and 18.5 years, respectively [21]. In Iran and Palestine, studies reported slightly higher ages of onset at 21.0 and 23.3 years, respectively [22,23], and in Jordan at 25.9 years [24]. In relation to gender, there is contradiction in the literature, with some studies reporting that men experience keratoconus more often than women [25,21], others reporting higher prevalence in women [26], and some showing no gender difference [23].

Diseases that affect vision, particularly those appearing at a young age such as keratoconus, clearly impact overall quality of life (QoL) [27]. Studies highlight issues related to functional dependence on third parties, social isolation, and limitations in daily activities [28]. Additionally, they demonstrate significant impairment in both near and distance vision, with direct effects on basic daily activities such as reading and driving. The progressive nature of keratoconus, even with non-surgical treatment, leads to gradual decreases in visual acuity, functional independence, and overall well-being [29,30]. Keratoconus significantly impairs visual function, causing blurring, distortions, and increased glare, which interfere with daily activities such as reading, recognizing faces, and using digital devices [31]. QoL studies report reduced scores in general vision, eye pain, near vision, vision-related mental health, role limitations, contrast sensitivity, and peripheral vision [32]. Symptoms including itching, redness, photophobia, and contact lens discomfort further limit functionality and negatively affect social interactions and self-image [33]. The disease also impacts education and early professional life, restricting school participation, career choices, and professional development [34], while social and recreational activities are often reduced, with emotional distress alleviated only by family or social support [35].

Keratoconus imposes substantial economic burdens through direct costs, such as corneal transplantation and ongoing care, and indirect costs, including work absenteeism and caregiver support [36-39]. Estimated annual or lifetime costs vary internationally, from AU$3,365 per patient in Australia [40] to US$24,000-29,000 in the United States [41,42], and US$2,342 per year in Saudi Arabia [37]. Limited insurance coverage further exacerbates the financial impact [40,43].

The primary aim of this study was to evaluate the impact of keratoconus on vision-related QoL (VRQoL) and work productivity. Secondary aims were to assess activity impairment and explore their associations with demographic characteristics, disease severity, treatment, comorbidities, and economic burden.

## Materials and methods

Procedure

The study included patients with keratoconus who visited the outpatient clinics of the First Department of Ophthalmology of the General Hospital of Athens following a medical appointment between February 2023 and February 2025. The study was conducted at the specialized Cornea Department of this university-affiliated clinic, which functions as a tertiary ophthalmology referral center serving patients from central and southern Greece.

Eligibility criteria and sampling

Participants were eligible for inclusion if they had a confirmed diagnosis of keratoconus and were aged 18 years or older. Patients were excluded if they had other ocular diseases that could significantly affect visual function (e.g., advanced glaucoma or retinal disease) or if they were unable to complete the questionnaires.

A convenience sampling strategy was applied, whereby all eligible patients attending the outpatient clinic during the study period were invited to participate. The disease was then staged by a specialist physician according to the Amsler-Krumeich classification, which divides keratoconus into Stages 1, 2, 3, and 4, corresponding to incipient, moderate, severe, and advanced keratoconus [44]. Eligible patients were approached immediately after their clinical examination and invited to participate in the study. Those who agreed provided written informed consent prior to data collection.

Ethics approval

The study was conducted in accordance with the principles of the Declaration of Helsinki. Ethical approval was obtained from the Bioethics Committee of the Medical School, National and Kapodistrian University of Athens (approval 568/19-11-2021).

Tools/measurements

Data in this study were collected using a structured, study-specific questionnaire designed to capture multiple dimensions relevant to keratoconus and its impact. The questionnaire was administered anonymously and consisted of sections covering participants’ demographic, social, medical, and economic characteristics, allowing a comprehensive understanding of the sample profile and potential confounding factors.

VRQoL was assessed using the Visual Function Questionnaire-25 (VFQ-25), developed by the National Eye Institute and widely employed in clinical and epidemiological research [45]. The VFQ-25 evaluates multiple domains, including general vision, ocular pain, near and distance activities, social functioning, mental health, role difficulties, dependency, and driving, and has been demonstrated to be a reliable and valid tool. Each subscale is scored on a 0-100 scale, with higher values indicating better outcomes; a score of 100 reflects optimal status, whereas 0 reflects the poorest possible status. It has been validated and culturally adapted for the Greek population and is freely available for research use [46].

The impact of keratoconus on work productivity and daily activities was assessed using the Work Productivity and Activity Impairment (WPAI) Questionnaire, which measures absenteeism (i.e., work time missed), presenteeism (i.e., impairment while working), overall work productivity loss (i.e., combination of absenteeism and presenteeism), and impairment in regular activities outside of work [47]. Higher scores reflect worse outcomes in terms of absenteeism, presenteeism, and overall productivity, indicating a higher level of impairment. The WPAI has also been validated for use in Greek populations and is freely available for research purposes [48].

Keratoconus severity was graded separately for each eye (OD: right eye; OS: left eye) according to the Amsler-Krumeich classification [44]. This classification system categorizes the disease into four stages (I-IV) based on refractive error (myopia and astigmatism), mean keratometry readings, corneal thickness (pachymetry), and the presence or absence of corneal scarring. Staging was performed by an ophthalmology resident in collaboration with a professor of ophthalmology to ensure diagnostic accuracy and consistency.

Staging of keratoconus was performed using a combined approach incorporating an autorefractometer, slit-lamp biomicroscopy, and corneal tomography with the Pentacam HR system (OCULUS GmbH, Wetzlar, Germany) to ensure a comprehensive assessment of the disease. Objective refraction parameters were recorded using the autorefractometer, with particular emphasis on irregular astigmatism. A slit-lamp examination was conducted to identify characteristic clinical signs, including the presence of Fleischer ring, Vogt’s striae, and corneal thinning. Quantitative evaluation was primarily based on Pentacam HR measurements, from which parameters such as maximum keratometry (Kmax), thinnest corneal pachymetry, and elevation maps were obtained, along with composite ectasia indices (e.g., BAD-D). Final staging was determined through the integration of these findings, according to established classification systems, including the Amsler-Krumeich classification, allowing for reliable categorization of keratoconus severity.

Together, these tools provided a multidimensional assessment of the functional, psychosocial, occupational, and economic burden of keratoconus, enabling a patient-centered analysis of its real-world impact.

Statistical analysis

All statistical analyses were performed using IBM SPSS Statistics for Windows, version 29.0.2.0, (released 2013; IBM Corp., Armonk, NY, USA). Categorical variables are presented as absolute and relative frequencies (%). Continuous variables are expressed as mean ± SD when normally distributed, and comparisons were conducted using independent samples t-tests or one-way ANOVA. For non-normally distributed variables, values are reported as median and IQR, and the Mann-Whitney U or Kruskal-Wallis H test was applied. Correlations between variables were assessed using Pearson correlation coefficients or Spearman’s rho where appropriate. Post hoc analyses were performed with Sidak or Games-Howell corrections for multiple comparisons, depending on data distribution. Finally, a multinomial linear regression analysis was conducted using the enter method.

For the estimation of total annual out-of-pocket cost included in the multiple linear regression analysis, a composite cost variable was calculated using the following formula:

$\mathrm{Cost} = A + (12 \times B) + C + (D \times E)$

Variable A represented the amount paid out-of-pocket for ophthalmology visits without reimbursement. Variable B corresponded to the monthly out-of-pocket expenditure for medications and diagnostic tests related to the condition, which was annualized by multiplying by 12. Variable C reflected any out-of-pocket payment for surgical intervention, when applicable. Variable D indicated the frequency of hospital visits for follow-up during the previous 12 months, and variable E represented the estimated transportation cost per hospital visit (round trip). The total cost variable derived from this formula was subsequently used as the dependent variable in the multiple linear regression model.

Comorbidity was defined as the presence of at least one self-reported chronic systemic or ocular condition other than keratoconus. The type of treatment was categorized as conservative (e.g., spectacles or contact lenses) or surgical (e.g., corneal cross-linking, intracorneal ring segments, or keratoplasty) based on the patient’s treatment history.

## Results

Demographic characteristics of the sample

A total of 102 participants were included in the study. Regarding gender distribution, 71.6% (n = 73) were male, while 28.4% (n = 29) were female (Table 1). Age was categorized into equal groups: ≤25, 26-35, and ≥36 years (n = 34, 33.3% for each category).

**Table 1:** Demographic characteristics of the sample (N = 102) Values are presented as absolute numbers and relative frequencies (%).

Characteristics	n	%
Gender		
Male	73	71.6
Female	29	28.4
Educational level		
Not at all, few primary school classes	3	3
Primary school graduate	1	1
Secondary school graduate	8	7.9
High school graduate	36	35.6
Graduate/diploma holder of college	8	7.9
Bachelor’s degree	34	33.7
Master’s/PhD degree	11	10.9
Job		
Public sector employee	8	8.1
Private sector employee	37	37.4
Freelancer	19	19.2
Unpaid employee in a family business	1	1
Unemployed/temporarily out of work	11	11.1
Retired	3	3
Student/housewife	20	20.2
Residence		
Attica	54	52.9
Peloponnese	23	22.5
Central Greece	9	8.8
Aegean	6	5.9
Crete	4	3.9
Thessaly	2	2
Thrace	3	2.9
Macedonia	1	1
Age (years)		
≤25	34	33.3
26-35	34	33.3
36+	34	33.3

Regarding educational level, three participants (3.0%) had attended a few primary school classes, one (1.0%) had graduated from primary school, eight (7.9%) had graduated from secondary junior high school, 36 (35.6%) from high school, eight (7.9%) were diploma holders from college, 34 (33.7%) had a bachelor’s degree, and 11 (10.9%) had a master’s/PhD degree. Regarding employment status, the majority were private sector employees (37.4%), freelancers (19.2%), and students/housewives (20.2%). According to patients’ residency, over 50% of participants lived in the Attica region (52.9%), followed by those from the Peloponnese region (22.5%).

Impact of vision function on work impairment

Correlation analysis was conducted to explore the relationship between the VFQ-25 and WPAI subscales (Table 2).

**Table 2:** Correlation analysis of the study variables ^*^ p < 0.05 ^** ^p < 0.01 ^***^ p < 0.001 Values are expressed as Pearson’s and ^†^Spearman’s ρ correlation coefficients.

Variables	Absenteeism	Presenteeism	Work productivity loss	Activity impairment
General health	-0.239^*^	-0.370^**^	-0.370^**^	-0.412^***^
General vision	-0.177	-0.085	-0.101	-0.217^*^
Ocular pain	-0.102	-0.338^**^	-0.308^**^	-0.357^***^
Near activities	-0.187	-0.460^***^	-0.431^***^	-0.421^***^
Distance activities	-0.124	-0.531^***^	-0.477^***^	-0.532^***^
Social functioning	-0.244^*,†^	-0.328^**,†^	-0.288^*,†^	-0.325^**,†^
Mental health	-0.181	-0.434^***^	-0.403^**^	-0.453^***^
Role differences	-0.207	-0.450^***^	-0.438^***^	-0.474^***^
Dependency health	-0.123	-0.428^***^	-0.388^**^	-0.405^***^
Driving	-0.174^†^	-0.359^*,†^	-0.303^*,†^	-0.442^**,†^
Color vision	-0.231^†^	-0.397^**,†^	-0.366^**,†^	-0.338^**,†^
Peripheral vision	-0.03	-0.468^***^	-0.413^***^	-0.525^***^
Total score	-0.174	-0.528^***^	-0.487^***^	-0.526^***^

The results revealed significant negative correlations between absenteeism and general health (r = -0.239, p < 0.05) as well as social functioning (rs = -0.244, p < 0.05). These findings suggest that poorer self-reported general health and reduced vision-related social functioning are associated with increased work absenteeism. Additionally, significant correlations were observed between presenteeism and general health (r = -0.370), ocular pain (r = -0.338), near activities (r = -0.460), distance activities (r = -0.531), social functioning (rs = -0.328), mental health (r = -0.434), role difficulties (r = -0.450), dependency (r = -0.428), driving (rs = -0.359), color vision (rs = -0.397), peripheral vision (r = -0.468), and total score (r = -0.528). Similar outcomes were observed between work productivity loss and VFQ-25 subscales (all significant except general vision). In contrast, significant correlations were observed between activity impairment and all VFQ-25 subscales (general vision included).

Comparisons were conducted to examine differences in VFQ-25 subscales across demographic and clinical characteristics of the sample (Table 3).

**Table 3:** Differences between VFQ-25 variables and demographic and clinical characteristics of the sample Values are presented as mean ± SD and analyzed using the t-test or ANOVA. ^†^ Values are presented as median and IQR and analyzed using the Mann-Whitney U test. Post hoc analysis was performed using Sidak or Games-Howell tests: ^a^ ≤25 vs. 26-35, ^b^ ≤25 vs. ≥36, ^c^ 26-35 vs. ≥36, ^d^ Stage 1 vs. 3, ^e^ Stage 2 vs. 3, and ^f^ Stage 2 vs. 4. Statistically significant differences are marked in bold. VFQ-25, Visual Function Questionnaire-25

Characteristic	General health	General vision	Ocular pain	Near activities	Distance activities	Social functioning	Mental health	Role differences	Dependency health	Driving	Color vision	Peripheral vision	Total score
Gender													
Male	36.25 ± 11.79	42.50 (10.50)	69.52 ± 15.45	72.26 ± 16.13	68.61 ± 17.18	81.37 ± 16.58	71.87 ± 16.77	70.32 ± 18.11	80.31 ± 18.09	75.00 (25.00)	100.00 (25.00)	72.26 ± 22.65	69.13 ± 14.19
Female	32.83 ± 11.71	33.00 (10.00)	63.79 ± 17.15	67.53 ± 14.91	61.95 ± 16.34	72.70 ± 23.87	60.56 ± 18.66	64.01 ± 17.49	69.18 ± 22.34	58.33 (8.33)	75.00 (25.00)	69.83 ± 20.46	62.33 ± 14.03
t/U^†^	1.326	798.50^†^	1.637	1.365	1.788	1.789	2.974	1.602	2.616	135.00^†^	884.00^†^	0.502	2.189
P	0.188	0.047^†^	0.105	0.175	0.077	0.081	0.004	0.112	0.01	0.084^†^	0.296^†^	0.617	0.031
Age (years)													
≤25	41.12 ± 12.24	40.18 ± 5.91	73.90 ± 13.54	78.19 ± 11.15	74.78 ± 14.47	86.76 ± 13.31	73.82 ± 14.83	72.61 ± 18.34	82.35 ± 17.23	76.11 ± 14.04	88.97 ± 15.31	83.82 ± 14.93	73.68 ± 10.94
26-35	35.72 ± 9.63	35.01 ± 6.59	66.91 ± 15.66	70.96 ± 17.15	66.89 ± 16.87	80.88 ± 19.41	69.15 ± 17.89	67.83 ± 18.28	79.04 ± 19.21	71.74 ± 14.16	87.50 ± 17.68	75.00 ± 21.32	67.83 ± 13.76
36+	29.00 ± 10.41	37.21 ± 8.06	62.87 ± 17.27	63.60 ± 15.57	58.48 ± 16.36	68.69 ± 20.20	62.98 ± 19.72	65.13 ± 17.34	70.04 ± 21.58	56.48 ± 24.68	72.66 ± 21.40	55.88 ± 19.52	60.07 ± 15.19
F	10.71	4.777	4.37	8.209	8.898	8.878	3.251	1.505	3.661	5.591	7.998	19.661	8.803
P	<0.001^b,c^	0.010^a^	0.015^b^	0.001^b^	<0.001^b^	<0.001^b^	0.043^b^	0.227	0.029^b^	0.006^b,c^	0.001^b,c^	<0.001^b,c^	<0.001^b^
OD Amsler-Krumeich stage													
1	40.08 ± 11.28	38.62 ± 5.86	75.00 ± 11.41	79.49 ± 9.54	74.42 ± 15.86	85.26 ± 16.01	76.73 ± 12.89	75.48 ± 14.08	86.06 ± 13.30	76.19 ± 15.54	91.67 ± 12.31	76.92 ± 16.01	74.55 ± 8.68
2	38.45 ± 10.06	38.90 ± 6.15	68.88 ± 15.96	73.55 ± 13.86	69.97 ± 17.11	82.47 ± 17.97	71.79 ± 16.26	72.32 ± 19.26	81.63 ± 19.24	72.53 ± 12.62	86.46 ± 17.83	75.51 ± 21.34	70.24 ± 13.97
3	28.71 ± 13.42	34.82 ± 6.67	65.18 ± 16.39	63.39 ± 18.29	55.95 ± 15.82	63.10 ± 23.28	53.57 ± 22.31	60.86 ± 18.60	65.63 ± 25.09	58.33 ± 18.00	67.86 ± 22.85	58.93 ± 23.22	57.24 ± 16.53
4	26.89 ± 15.27	32.11 ± 11.18	58.33 ± 18.75	65.28 ± 20.94	61.67 ± 20.67	77.78 ± 21.65	64.44 ± 16.67	59.03 ± 15.97	68.06 ± 18.34	69.44 ± 19.25	83.33 ± 21.65	66.67 ± 25.00	61.37 ± 15.40
F	5.093	3.361	2.186	3.404	3.518	4.258	5.395	2.916	3.967	2.794	4.502	2.606	4.836
P	0.003^e,f^	0.023^f^	0.096	0.022^d^	0.019^d,e^	0.008^d,e^	0.002^d,e^	0.039^d,e^	0.011^d,e^	0.052	0.006^d^	0.057	0.004^d,e^
OS Amsler-Krumeich stage													
1	43.85 ± 10.63	40.35 ± 5.47	76.25 ± 12.43	78.75 ± 11.69	77.08 ± 15.62	88.33 ± 11.92	75.00 ± 13.94	75.00 ± 15.87	85.00 ± 14.19	78.33 ± 18.26	92.50 ± 16.87	87.50 ± 13.18	75.54 ± 10.54
2	36.38 ± 10.17	37.53 ± 5.77	68.11 ± 15.11	73.81 ± 11.69	70.56 ± 14.96	82.99 ± 16.48	72.50 ± 16.75	70.96 ± 19.31	80.74 ± 18.79	71.61 ± 12.32	86.46 ± 17.07	74.49 ± 20.08	69.99 ± 13.08
3	29.15 ± 9.51	33.75 ± 6.16	67.50 ± 22.97	60.00 ± 21.89	52.08 ± 16.23	62.50 ± 22.99	53.50 ± 19.73	67.50 ± 19.72	66.88 ± 28.11	64.58 ± 20.83	67.50 ± 20.58	57.50 ± 23.72	56.92 ± 17.92
4	31.06 ± 19.74	35.39 ± 12.74	68.06 ± 19.87	70.83 ± 22.05	66.76 ± 23.70	82.41 ± 25.15	65.00 ± 18.03	61.11 ± 16.76	73.61 ± 17.34	47.22 ± 19.25	83.33 ± 25.00	63.89 ± 25.34	65.29 ± 17.51
F	3.258	1.779	0.724	3.177	4.569	4.248	4.032	1.017	1.948	3.473	3.616	4.245	3.452
P	0.026^d^	0.159	0.541	0.029^d,e^	0.005^d,e^	0.008^d,e^	0.010^d,e^	0.39	0.129	0.025^d,e^	0.017^d,e^	0.008^d^	0.021^d^
Type of treatment													
Conservative	37.26 ± 11.06	37.74 ± 6.99	70.47 ± 15.83	74.50 ± 14.50	69.05 ± 17.34	79.68 ± 19.80	70.17 ± 17.45	72.09 ± 18.33	79.31 ± 19.91	75.25 ± 12.89	84.82 ± 18.88	74.57 ± 21.71	69.60 ± 14.23
Surgical	32.00 ± 12.17	36.82 ± 7.51	64.29 ± 16.23	66.07 ± 16.88	63.29 ± 16.87	77.78 ± 18.92	66.49 ± 19.07	63.44 ± 17.00	74.26 ± 20.30	55.95 ± 23.30	81.55 ± 19.95	66.67 ± 21.86	63.77 ± 14.44
t	2.25	0.629	1.909	2.676	1.658	0.481	1.002	2.4	1.243	3.959	0.822	1.792	2.011
P	0.027	0.531	0.059	0.009	0.101	0.632	0.319	0.018	0.217	<0.001	0.413	0.076	0.047
Comorbidity													
No	39.25 ± 10.15	38.28 ± 5.90	70.56 ± 14.82	74.18 ± 13.49	70.89 ± 15.43	83.66 ± 17.45	72.60 ± 16.00	71.16 ± 16.79	81.25 ± 17.14	71.63 ± 14.50	88.16 ± 16.06	77.63 ± 18.96	70.98 ± 11.90
Yes	25.36 ± 6.53	36.59 ± 9.61	63.07 ± 16.13	64.20 ± 17.52	57.80 ± 14.15	67.46 ± 15.12	61.14 ± 16.42	63.92 ± 19.66	69.03 ± 21.17	59.72 ± 29.05	71.25 ± 20.32	55.68 ± 21.73	59.14 ± 14.12
t	6.055	0.781	2.047	2.847	3.564	3.869	2.943	1.717	2.788	1.372	3.956	4.625	3.941
P	<0.001	0.442	0.043	0.005	0.001	<0.001	0.004	0.09	0.006	0.194	<0.001	<0.001	<0.001

Regarding gender, male participants reported higher general vision scores compared to females (median = 42.50 vs. 33.00, p = 0.047), mental health (mean ± SD = 71.87 ± 16.77 vs. 60.56 ± 18.66, p = 0.004), dependency (mean ± SD = 80.31 ± 18.09 vs. 69.18 ± 22.34, p = 0.010), and total VFQ-25 score (mean ± SD = 69.13 ± 14.19 vs. 62.33 ± 14.03, p = 0.031). In terms of age, significant differences were observed across age groups in all VFQ-25 subscales except role difficulties. Generally, patients aged 36 years and older reported significantly lower scores.

In OD Amsler-Krumeich stage, significant differences were observed across the four groups in all VFQ-25 subscales except ocular pain, driving, and peripheral vision. Generally, patients with higher OD Amsler-Krumeich stages had significantly lower scores. Similarly, in OS Amsler-Krumeich stage, significant differences were observed across the four groups in all VFQ-25 subscales except general vision, ocular pain, role difficulties, and dependency. Generally, patients with higher OS Amsler-Krumeich stages had significantly lower scores.

Regarding the type of treatment, significant differences were observed across the VFQ-25 subscales of general health, near activities, role difficulties, driving, and total score; higher scores were observed in the conservative treatment group. Differences in comorbidity were observed in all VFQ-25 subscales except general vision, role difficulties, and driving; lower scores were observed in patients with comorbidity.

Impact of demographic characteristics on work impairment

Table 4 presents the differences in WPAI variables according to demographic and clinical characteristics of the study sample. No statistically significant differences were observed between males and females across absenteeism, presenteeism, overall work productivity loss, and activity impairment (p > 0.05).

**Table 4:** Differences between WPAI variables and demographic and clinical characteristics of the sample Values are reported as mean ± SD, and comparisons were performed using the t-test or ANOVA. ^†^ Values are presented as median and IQR and analyzed using the Mann-Whitney U test or Kruskal-Wallis H test. Post hoc analyses (Sidak or Games-Howell) were performed for^ a^ ≤25 vs. 26-35,^ b^ ≤25 vs. 36+, ^c^ 26-35 vs. 36+, ^d^ Stage 1 vs. 4, and ^e^ Stage 2 vs. 4. Statistically significant differences are marked in bold. WPAI, Work Productivity and Activity Impairment Questionnaire

Characteristic	Absenteeism	Presenteeism	Work productivity loss	Activity impairment
Gender				
Male	9.83 ± 19.82	43.20 ± 26.84	47.54 ± 28.16	46.71 ± 28.63
Female	13.78 ± 22.12	49.05 ± 21.89	53.80 ± 23.65	52.41 ± 18.83
t	-0.738	-0.959	-0.957	-1.177
P	0.463	0.343	0.344	0.243
Age (years)				
≤25	6.25 (11.67)	39.47 ± 25.71	43.61 ± 26.63	37.65 ± 24.13
26-35	3.00 (10.61)	40.34 ± 27.58	44.18 ± 28.50	46.76 ± 28.68
36+	4.76 (5.87)	55.22 ± 19.74	60.61 ± 22.20	60.59 ± 20.74
F/H^†^	2.828^†^	2.938	3.156	7.418
P	0.243^†^	0.06	0.049^b,c^	<0.001^a^
OD Amsler-Krumeich stage				
1	3.23 (5.63)	37.78 ± 24.89	40.04 ± 24.89	44.62 ± 21.06
2	4.76 (5.90)	40.00 ± 25.00	44.32 ± 25.97	45.10 ± 28.44
3	7.25 (16.89)	56.67 ± 22.70	60.52 ± 23.48	54.29 ± 22.09
4	9.09 (59.38)	52.00 ± 20.49	65.41 ± 27.90	51.11 ± 29.77
F/H^†^	5.108^†^	1.772	2.24	0.536
P	0.164^†^	0.163	0.094	0.659
OSAmsler-Krumeich stage				
1	3.23 (2.69)	26.00 ± 23.02	28.11 ± 23.02	29.00 ± 21.32
2	4.76 (7.93)	41.05 ± 26.49	46.36 ± 28.95	46.12 ± 28.20
3	4.32 (14.88)	45.00 ± 24.29	47.60 ± 26.14	59.00 ± 26.01
4	7.25 (18.20)	67.50 ± 12.58	71.01 ± 11.57	65.56 ± 12.36
F/H^†^	1.879^†^	2.058	1.818	3.864
P	0.598^†^	0.118	0.156	0.013^d,e^
Type of treatment				
Conservative	5.26 (9.09)	40.00 ± 24.78	44.50 ± 26.52	42.76 ± 26.87
Surgical	4.65 (13.05)	53.33 ± 25.12	58.20 ± 26.04	56.67 ± 23.86
t/U^†^	528.00^†^	-2.18	-2.093	-2.676
P	0.699^†^	0.033	0.04	0.009
Comorbidity				
No	4.65 (8.78)	42.08 ± 24.76	46.60 ± 25.92	44.74 ± 26.10
Yes	4.76 (11.51)	48.67 ± 26.42	53.11 ± 29.34	55.00 ± 24.25
t/U^†^	350.50^†^	-0.897	-0.832	-1.649
P	0.550^†^	0.373	0.409	0.102

When stratified by age, older participants (≥36 years) reported significantly higher levels of work productivity loss (p = 0.049) and activity impairment (p < 0.001) compared to younger age groups. A similar trend was observed with disease severity. In particular, higher Amsler-Krumeich stages of the worse-seeing eye (OS) were associated with greater activity impairment (p = 0.013), with post hoc comparisons indicating significant differences between Stage 1 and Stage 4, as well as between Stage 2 and Stage 4.

Regarding type of treatment, patients who had undergone surgical intervention demonstrated significantly greater presenteeism (p = 0.033), overall work productivity loss (p = 0.040), and activity impairment (p = 0.009) compared with those receiving conservative treatment, whereas absenteeism did not differ significantly between the groups. The presence of comorbidities was not associated with statistically significant differences in any of the WPAI domains (all p > 0.05).

Multinomial linear regression analysis of cost and VFQ-25 subscales

Table 5 summarizes the results of the multinomial linear regression analysis, in which cost was modeled as the dependent variable and the subscales of the VFQ-25 were entered as independent predictors. None of the VFQ-25 domains demonstrated statistically significant associations with cost (all p > 0.05).

**Table 5:** Multinomial linear regression with cost (€ per year) as the dependent variable and VFQ-25 variables as independent VFQ-25, Visual Function Questionnaire-25

Variables	β	P	95% CI
General health	-28.32	0.744	-202.31, 145.67
General vision	132.83	0.397	-180.00, 445.66
Ocular pain	50.09	0.511	-102.32, 202.50
Near activities	41.75	0.645	-139.70, 223.19
Distance activities	-75.37	0.41	-257.90, 107.17
Social functioning	136.18	0.133	-43.00, 315.35
Mental health	8.79	0.934	-205.44, 223.02
Role differences	35.96	0.605	-103.06, 174.99
Dependency health	-25.55	0.805	-232.68, 181.58
Driving	25.41	0.611	-74.63, 125.46
Color vision	-102.15	0.14	-239.18, 34.89
Peripheral vision	-18.04	0.743	-128.51, 92.43

Although not reaching statistical significance, social functioning (β = 136.18, p = 0.133, 95% CI: -43.00 to 315.35) and color vision (β = -102.15, p = 0.140, 95% CI: -239.18 to 34.89) showed the largest effect estimates in opposite directions, suggesting potential trends that warrant further exploration. All other subscales, including general health, general vision, ocular pain, near and distance activities, mental health, role difficulties, dependency, driving, and peripheral vision, exhibited weak and non-significant relationships with cost.

## Discussion

This study investigated the impact of keratoconus on VRQoL, work productivity, and the economic cost of the disease, highlighting the multidimensional burden experienced by patients. The findings confirm that keratoconus is not only an ophthalmological condition but also a chronic disease with significant functional, psychosocial, and occupational impacts.

The majority of participants were men, who reported higher scores on the VFQ-25 subscales, particularly in general vision, mental health, dependency, and the VFQ-25 total score, compared to women. This finding is consistent with some studies indicating better perceived functioning in male patients [25,21,49]; however, other studies report no gender differences [23] or indicate lower QoL in female patients [32]. These differences are likely influenced by sociocultural factors, gender roles, and variations in symptom perception and reporting.

Age emerged as a significant determinant of burden, with older patients (≥36 years) showing significantly lower VRQoL and increased productivity loss. This finding aligns with the long-term nature of keratoconus and the cumulative impact of the disease on visual function, as highlighted in population studies and cost analyses [5,50]. Additionally, deterioration of VRQoL with age may be associated with increased occupational and family obligations, making functional losses more apparent [29,30].

According to the Amsler-Krumeich staging system, higher grades in both the right (OD) and left (OS) eyes were associated with significantly lower QoL. This can be interpreted in light of the fact that greater structural damage leads to increased discomfort and functional limitations. Therefore, the subjective experience of the disease is largely determined by the functional status of the eye with lower visual performance, as reported in previous studies [32,33]. A study in France similarly demonstrated that reduced distance-corrected visual acuity in the better-seeing eye was the main factor predicting lower VRQoL [31]. These results underscore the importance of early detection and timely intervention in the initial stages of the disease.

Patients who underwent surgical treatment had lower VRQoL and greater loss of productivity compared to those receiving conservative treatment. Similar results have been reported in studies where surgical interventions were primarily performed in patients with greater clinical severity and long-term disease progression [36,38]. The immediate postoperative period often involves temporary discomfort and reduced visual acuity, resulting in a transient decrease in VRQoL, as noted by Marx-Gross et al. [51].

Pinto et al. demonstrated improvement in VRQoL after surgery, particularly in subscales related to activity limitations and symptoms [52]. However, surgery usually does not reverse existing corneal lesions, and patients may still require contact lenses or other interventions to correct vision. Consequently, VRQoL scores may not improve substantially, suggesting that the psychological burden of the disease may persist even after successful treatment [53]. Therefore, these findings reflect the severity of the underlying disease more than the effectiveness of the surgery itself.

A key finding of this study is the consistent and strong negative correlation between VFQ-25 subscales and WPAI indices. Worse self-reported general health and reduced vision-related social functioning were associated with reduced work productivity. These results suggest that reduced perceived visual function is more strongly related to decreased performance while at work (presenteeism) rather than complete absence from work [30,34,54].

Keratoconus is associated with a significant reduction in overall work productivity and limitations in daily activities. Strong correlations were observed across multiple VRQoL subscales, including general health, social functioning, mental health, near and distance vision tasks, and driving. These findings indicate that the functional impact of keratoconus extends beyond visual acuity loss, substantially affecting both daily life and professional performance.

Presenteeism is characterized by attending work with reduced performance, increasing the risk of errors and potential harm to health. Limited annual leave, workplace pressure, and company cultures that do not promote employee well-being exacerbate this burden. Studies have shown that difficulty in reading and driving and increased visual fatigue directly affect professional performance and increase psychological stress in patients [31,32,46]. The present study reinforces the view that VRQoL is a critical predictor of functional capacity and should be considered in the design of interventions and health policies. Reduced VRQoL, particularly in general health and social functioning, contributes to increased work burden, especially since keratoconus primarily affects young, working-age individuals. In this study, no VFQ-25 subscale was independently associated with the annual economic cost of keratoconus. This suggests that subjective perceived QoL may not be directly associated with the economic burden of the disease within this study’s context.

International literature indicates that the economic cost of keratoconus primarily depends on the type of therapeutic intervention, frequency of follow-up in specialized centers, and reimbursement policies in each health system. VRQoL functions more as an indicator of functional burden than as a direct determinant of medical expenses [55]. Health systems with broader insurance coverage show a more homogeneous financial burden among patients with similar clinical profiles [56].

In the Greek health system, limited reimbursement for specialized contact lenses and modern interventions shifts costs to patients. Therefore, financial burden is influenced more by affordability, geographic access, and use of private health services than by visual function. The absence of an association between VFQ-25 subscales and costs may reflect inequalities in access to care rather than a lack of relationship between functional burden and health resource use. Our sample consisted of patients visiting a hospital in the capital, although most resided far away due to the absence of specialized centers in other regions, which further increased financial burden. Additionally, the VFQ-25, while valid for assessing QoL, does not directly capture healthcare utilization or direct and indirect economic costs. In Greece, where keratoconus often affects working-age adults, indirect costs from reduced productivity or limited occupational opportunities may be significant but not fully reflected in QoL measures or direct cost calculations.

Overall, the findings align with international literature and highlight that, in Greece, the economic burden of keratoconus is largely a matter of health policy and coverage. Policies that consider the chronic and progressive nature of the disease and emphasize coverage of timely and cost-effective interventions could reduce disparities, lower long-term financial burden, and substantially improve patient QoL. The study findings support the need for multidimensional, patient-centered approaches in keratoconus management. Incorporating VRQoL and productivity assessment tools into clinical practice can facilitate early identification of patients at high risk for functional and economic burden and inform targeted interventions, such as work support programs and improved access to specialized care [42,43]. Future prospective studies with larger samples and more detailed mapping of direct and indirect costs could enhance understanding of the overall burden of keratoconus and optimize health policy. Given the cross-sectional design, the observed relationships should be interpreted as associations rather than causal effects, and no conclusions can be drawn regarding directionality.

Limitations of the study

The present study has several limitations that should be considered when interpreting the findings. First, this was a single-center study, as the sample was recruited exclusively from a tertiary ophthalmology referral center in Athens. Therefore, the findings may not be fully generalizable to the broader population of patients with keratoconus, particularly those who do not have access to specialized healthcare services or are managed in primary care settings. Furthermore, the cross-sectional design does not allow for investigation of causal relationships between variables, limiting conclusions to associations. Consequently, no definitive conclusions can be drawn regarding the temporal relationship between disease severity and its impact on QoL or work productivity.

Despite these limitations, the study provides important insights into the multidimensional impact of keratoconus on QoL, work productivity, and the economic burden experienced by patients, thereby contributing to a better understanding of the real-world impact of the disease in the Greek population. Additionally, the study sample was derived from a tertiary referral center, which may introduce selection bias, as patients attending such facilities are more likely to present with advanced or complex forms of the disease. Therefore, the findings may not fully reflect the broader population of individuals with keratoconus, particularly those with milder disease managed in primary or secondary care settings.

Although multivariable analyses were performed, residual confounding cannot be excluded, as not all potential influencing factors (e.g., socioeconomic status, occupational characteristics, or access to healthcare services) were fully captured or adjusted for. Regarding the economic analysis, cost estimates were based on self-reported data and a composite cost variable, which may be subject to recall bias and may not fully capture indirect or intangible costs. In addition, no detailed stratification of costs by socioeconomic or insurance status was performed, which may limit the interpretation of economic burden across different population subgroups.

These limitations should be considered when interpreting the findings. Future multicenter, longitudinal studies with more detailed economic evaluation are warranted to confirm and extend the present results.

## Conclusions

Keratoconus is a complex, chronic condition whose impact extends far beyond visual impairment, profoundly affecting daily functioning, psychosocial well-being, and work productivity. Disease severity, age, and treatment modality are key determinants of VRQoL and occupational performance, whereas economic burden is primarily shaped by systemic factors and healthcare access rather than perceived QoL alone.

Notably, this study represents the first investigation in Greece to examine the relationship between VFQ-25 subscales and WPAI indices, highlighting the functional and occupational dimensions of the disease beyond visual acuity loss. These findings emphasize the critical importance of early diagnosis and patient-centered interventions. Integrating VRQoL and productivity assessments into routine clinical practice can facilitate the identification of high-risk patients, guide individualized management strategies, and inform health policy, ultimately reducing long-term disease burden and improving overall QoL for individuals with keratoconus.
